# Age Is a Predictor of In-Hospital Outcomes for Left Ventricular Assist Device Implantation: A Nationwide Analysis

**DOI:** 10.3390/jpm14030236

**Published:** 2024-02-22

**Authors:** Abdul Rahman Akkawi, Akira Yamaguchi, Junichi Shimamura, Omar Chehab, Paulino Alvarez, Toshiki Kuno, Alexandros Briasoulis

**Affiliations:** 1 Department of Internal Medicine, Kansas University School of Medicine-Wichita, Wichita, KS 67214, USA; aakkawi@kumc.edu; 2 Division of Cardiovascular Surgery, University of Tsukuba, Tsukuba 305-8577, Ibaraki, Japan; 3 Division of Cardiothoracic Surgery, Department of Surgery, Westchester Medical Center, Valhalla, NY 10595, USA; 4 Division of Cardiology, Department of Medicine, Johns Hopkins University Baltimore, Baltimore, MD 21218, USA; 5 Department of Cardiovascular Medicine, Cleveland Clinic, Cleveland, OH 44195, USA; 6 Division of Cardiology, Montefiore Medical Center, Albert Einstein College of Medicine, Bronx, NY 10461, USA; 7 Division of Cardiovascular Medicine, Section of Heart Failure and Transplantation, University of Iowa, Iowa City, IA 52242, USA

**Keywords:** left ventricular assist device, heart allocation system, in-hospital mortality

## Abstract

The 2018 heart allocation system has significantly influenced heart transplantation and left ventricular assist device (LVAD) utilization. Our study aims to investigate age-related outcomes following LVAD implantation in the post-allocation era. Using the National Inpatient Sample, we analyzed data from 7375 patients who underwent LVAD implantation between 2019 and 2020. The primary endpoint was in-hospital mortality following LVAD implantation, stratified by age categories. The age groups were 18–49, 50–59, 60–69, and over 70. These represented 26%, 26%, 31%, and 17% of patients, respectively. Patients aged 60–69 and those over 70 exhibited higher in-hospital mortality rates of 12% and 17%, respectively, compared to younger age groups (7% for 18–49 and 6% for 50–59). The age groups 60–69 and over 70 were independent predictors of mortality, with adjusted odds ratios of 1.99 (*p* = 0.02; 95% confidence interval [CI], 1.12–3.57) and 2.88 (*p* = 0.002; 95% CI, 1.45–5.71), respectively. Additionally, a higher Charlson Comorbidity Index was associated with increased in-hospital mortality risk (adjusted odds ratio 1.39; *p* = 0.02; 95% CI, 1.05–1.84). Additionally, patients above 70 experienced a statistically shorter length of stay. Nonhome discharge was found to be significantly high across all age categories. However, the difference in hospitalization cost was not statistically significant across the age groups. Our study highlights that patients aged 60 and above face an increased risk of in-hospital mortality following LVAD implantation in the post-allocation era. This study sheds light on age-related outcomes and emphasizes the importance of considering age in LVAD patient selection and management strategies.

## 1. Introduction

Heart transplant (HTx) is a definitive treatment for patients with end-stage heart failure; however, organ shortage has brought higher waitlist mortality rates [1]. 

Durable left ventricular assist device (LVAD) implantation has improved the waitlist mortality, [2] making 3684 LVAD patients HTx candidates from 2015 to 2020 [3]. However, under the old organ allocation system, 67% of adult HTx was performed in status 1A, and 36% of waitlisted patients had a durable VAD [4]. This overcrowding in status 1A highlighted the need for modified risk stratification.

In 2018, the Organ Procurement and Transplantation Network implemented a new organ allocation system to equitize the HTx opportunity based on medical urgency [5]. Consequently, stable, durable LVAD candidates are classified as status 4.

In the post-allocation era, the number of bridge-to-transplant (BTT) patients decreased, and their one-year post-transplant survival rates worsened due to the presence of more risk factors including age, ischemic etiology, renal function, functional status, obesity, and pulmonary hypertension (93.6% versus 87.3%) [6].

In the pre-allocation era, predictors of in-hospital mortality for LVAD implantation included age, hemodialysis, cerebrovascular disease, mechanical ventilation, liver disease, acute kidney injury (AKI), disseminated intravascular coagulation, sepsis, septic shock, and gastrointestinal bleeding [7]. However, HeartMate 3 (HM3; Abbott Cardiovascular, Chicago, IL, USA) has reduced complications, such as thrombosis and stroke events [8], and is the majority in the post-allocation era (47.3% versus 8.3%) [9]. This device improvement plausibly has changed predictors of in-hospital mortality and expanded the age demographic of patients receiving LVAD implantation. As the utilization of LVAD continues to rise, the financial implications, which include both costs and length-of-stay (LOS) associated with LVAD, have become increasingly significant for various stakeholders in healthcare [10]. We aimed to investigate in-hospital mortality, total hospital charges, discharge destination, and LOS of LVAD patients across age categories; we also explored the independent predictors of in-hospital mortality and LOS in the post-allocation era.

## 2. Materials and Methods

### 2.1. Data Source

This is an observational, retrospective study. The analysis utilized the National Inpatient Sample (NIS) of the Health Care Quality and Utilization Project, which represents a 20% sample of all inpatient discharges from various hospitals. NIS includes patients and hospital-level characteristics, mortality rates, in-hospital complications, and health utilization information. The sample design and description have been previously described, and these are available online [11]. Institutional review board approval was waived as the dataset is deidentified and publicly available, and does not involve the usage of test materials.

### 2.2. Patients and Variables

We identified patients aged over 18 who underwent LVAD implantation between January 2019 and December 2020 by using the International Classification of Diseases, Tenth Revision procedure code (ICD-10-PCS) “02HA0QZ”. Exclusion criteria for this study’s subjects were defined as patients under 18 years old and those with missing data on their comorbidities, including hypertension, chronic heart failure (CHF), coronary artery disease (CAD), dyslipidemia, previous myocardial infarction (MI), chronic obstructive pulmonary disease (COPD), liver disease, chronic kidney disease (CKD), peripheral vascular disease (PVD), previous coronary artery bypass grafting (CABG), and smoking status.

Data on patient- and hospital-level characteristics, LOS, discharge destination, total hospitalization charges, and comorbidities were extracted. Hospital resource information was obtained from the database, and comorbidity information was retrieved from ICD-10 codes. The patient comorbidity burden was assessed using the Charlson Comorbidity Index (CCI) [12].

### 2.3. Endpoints

The primary endpoint was in-hospital mortality in the following age categories: 18–49, 50–59, 60–69, and ≥70. In-hospital mortality was available as a categorical variable in the NIS data. The secondary endpoints were mean LOS, total hospitalization charges, discharge destination, and complications in age categories, including driveline infection, AKI, AKI requiring dialysis, ischemic stroke, postoperative bleeding events (intracranial hemorrhage, gastrointestinal bleeding, hemoptysis, hematuria, epistaxis, and unspecified bleeding) and septic shock. Rates of in-hospital mortality and complications between 2019 and 2020 were compared across age categories. Independent predictors of in-hospital mortality and LOS were examined.

### 2.4. Statistical Analysis

The analysis followed recommended statistical and research methodologies for the NIS data [13]. Chi-square tests compared baseline, hospital-level characteristics, and discharge destination among age groups for categorical variables. The adjusted Wald test was used for continuous variables such as age, LOS, and total hospitalization charges. Given the large population size of the weighted NIS, *t*-tests were used for comparing continuous variables. The database’s standardized sampling and weighting method provided by the Agency for Healthcare Research and Quality enabled national estimates to be made for the entire hospitalized population in the US. Rates of in-hospital mortality and major complications between 2019 and 2020 were compared among age categories using *t*-tests, with *p*-values indicating trend analysis. A logistic regression model calculated adjusted odds ratios (aORs) with 95% confidence intervals (CIs) for the risks of in-hospital mortality and LOS related to LVAD implantation. The multivariable logistic regression model adjusted for potential factors, including age, gender, race, admission type, hospital region, teaching status, bed size, insurance, income, hypertension, dyslipidemia, CHF, COPD, CKD, CAD, previous MI, diabetes mellitus type 2, obesity, PVD, liver disease, previous CABG, and CCI. The analysis was conducted using Stata 17.0 (StataCorp. 2021. Stata Statistical Software: Release 17. College Station, TX, USA: StataCorp LLC.). A two-sided *p* < 0.05 was considered statistically significant.

## 3. Results

A total of 7375 patients underwent LVAD implantation from 2019 to 2020. Baseline characteristics are summarized in Table 1. Of the patients, 1935 were aged 18–49, 1900 were aged 50–59, 2300 were aged 60–69, and 1240 were over 70. The prevalence of comorbidities varied significantly among different age groups, including CAD, dyslipidemia, previous MI, liver disease and PVD. Most comorbidities were more prevalent in patients over 60. Most patients in all age groups had a CCI ≥ 3. Higher age groups had a higher proportion of CCI ≥ 3.

Complications of LVAD implantation are summarized in Table 2. The in-mortality rate was highest in the over-70 age group (17%) and was lowest in the 50–59 age group (6%). The incidence of bleeding complications differed significantly, with over 20% in the 18–69 age groups; however, this was lowest in the over 70 age group (14%).

In-hospital mortality and post-LVAD implantation complications between 2019 and 2020 were examined (Table 3). In-hospital mortality increased in the 18–49 age group (5% versus 9.6%) and in the over 70 age group (16.1% versus 18.5%) without a significant difference for trend analysis. None of the other variables showed significant differences, except for AKI requiring dialysis in the over 70 age group (*p* = 0.02).

Independent predictors of mortality in patients who underwent LVAD implantation were examined (Table 4). In the 60–69 age group and in the over 70 age group, the aORs were 1.99 (*p* = 0.02; 95% confidence interval (CI), 1.12–3.57) and 2.88 (*p*= 0.002; 95% CI 1.45–5.71), respectively (the 18–49 age group as a control). CCI (aOR, 1.39; *p* = 0.02; 95% CI 1.05–1.84) was associated with an increased in-hospital mortality rate.

We also investigated the mean LOS for patients admitted for LVAD implantation across the different age categories. The mean LOS varied across age groups, with patients aged 18–49 experiencing an average stay of 39.1 days (SD: 36.3–41.9); those aged 50–59 staying 37.7 days (SD: 34.9–40.6); patients aged 60–69 staying a mean of 36.5 days (SD: 33.9–39.1); and patients aged 70 and above exhibiting a shorter mean LOS at 31.9 days (SD: 29.2–34.5) (Table 5). Note that the differences in LOS were statistically significant, with a *p*-value of 0.01.

Using adjusted multivariable logistic regression, our analysis identified significant predictors affecting the length of stay (LOS) for LVAD implantation patients. Notably, patients aged 70 and above were associated with a significant reduction in LOS (β = −6.1; *p* = 0.01; 95% CI: −1.1, −1.3), indicating a shorter hospital stay for this age group. Additionally, regional disparities were observed, with hospitalizations in the west showing a decreased LOS (β = −6; *p* = 0.03; 95% CI: −11.3, −0.7), and those in urban teaching hospitals associated with shorter LOS (β = −10.6; *p* < 0.01; 95% CI: −17.3, −4). Conversely, CCI demonstrated a positive significant correlation with LOS. Specifically, CCI 1 (β = 22.1; *p* < 0.01; 95% CI: 6.78, 37.43), CCI 2 (β = 21.1; *p* < 0.01; 95% CI: 6.2, 36), and CCI 3 (β = 27.7; *p* < 0.01; 95% CI: 12.6, 42.8).

We also explored the total charges for patients undergoing LVAD implantation across age categories (Table 6). Patients aged 18–49 had a mean total charge of $1.25 (in units of 10^5^, SD: $1.13–$1.35), those in the 50–59 age group had a mean total charge of $1.14 (10^5^, SD: $1.04–$1.23), and patients aged 60–69 experienced a mean total charge of $1.17 (10^5^, SD: $1.09–$1.26). Notably, individuals aged 70 and above had a mean total charge of $1.11 (in units of 10^5^, SD: $1.01–$1.21). However, there was no significant difference in the total charges between the age categories, with a *p*-value of 0.23.

**Figure 1:**
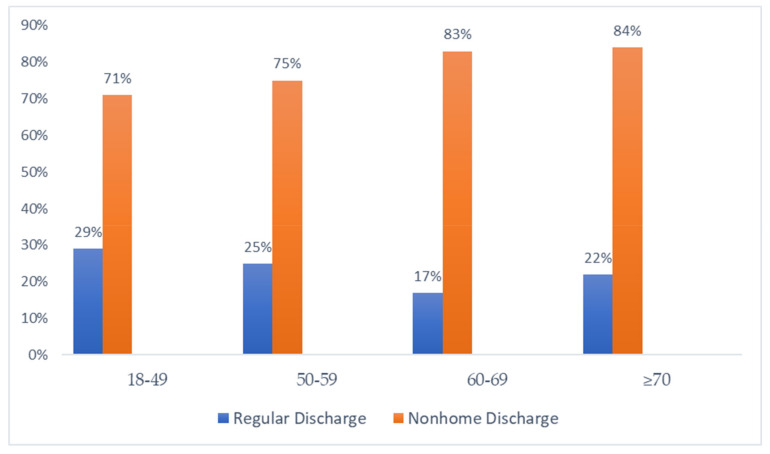
Post–hospitalization destinations of patients following LVAD implantation, *p*-value < 0.01.

Figure 1 shows post-hospitalization destinations for LVAD implantation patients who are discharged alive. Nonhome discharge, including short-term hospitals, skilled nursing facilities, intermediate care, and home health care, showed rates of 71%, 75%, 83%, and 84% for age groups 18–49, 50–59, 60–69, and ≥70, respectively. The *p*-value (<0.01) indicates a statistically significant higher rate of nonhome discharge compared to regular discharge.

## 4. Discussion

We analyzed 7375 patients from the NIS database who underwent LVAD implantation in the post-allocation era. In-hospital mortality rates for post-LVAD implantation were 7%, 6%, 12%, and 17% in the 18–49, 50–59, 60–69, and ≥70 age groups, respectively. Independent predictors of in-hospital mortality included being over 60 years old and higher CCI.

### 4.1. Device and Surgical Techniques Improvement

First-generation LVADs had limitations due to their larger size, limited durability, higher rates of infections, and device failure. Continuous flow LVADs emerged as superior alternatives with smaller size, enhanced durability, reduced infection rates, and decreased bleeding incidents [14,15]. Currently, HM3 demonstrates better outcomes compared to HeartMate II (Abbott Cardiovascular, Chicago, IL, USA), including in survival rates, pump thrombosis, device exchanges, stroke, and gastrointestinal bleeding [16,17]. Additionally, minimally invasive surgery, such as left lateral thoracotomy combined with upper mini-sternotomy or upper right thoracotomy, have shown advantages over full sternotomy, including mortality rate, postoperative drainage volume, and usage of blood products [18].

### 4.2. Organ Availability and Patients’ Status

The new allocation system has impacted organ availability. The number of HTx for LVAD patients decreased between 2016 and 2020, and post-transplant survival worsened due to the acceptance of marginal donors [19]. However, in the post-allocation era, the total number of adult HTx increased from 2945 to 3032, and approximately 35% of all candidates were in statuses 1–3, compared to 25% in the pre-allocation era. Moreover, there were more candidates with extracorporeal membrane oxygenation (ECMO) or intra-aortic balloon pumping (IABP) support in post-allocation system (ECMO, 1.02% vs. 5.28%; IABP, 7.48% vs. 27.0%), suggesting prioritization of organs for patients with urgent conditions. Additionally, the number of BTT patients and the median waitlist-to-transplantation times decreased [4,9,20].

In the post-allocation era, the increased distance between recipients and donors led to a longer ischemic time until transplantation in all candidates (3.0 ± 1.0 versus 3.4 ± 0.96 h), resulting in higher 180-day post-transplant mortality rates (77.9% versus 93.4%) [21]. Moreover, LVAD recipients of HTx had worse functional status with higher hospitalization rates in the post-allocation era (13.6% versus 22.4%) [19].

### 4.3. Shift from BTT to DT

While previous studies have suggested worse outcomes for HTx in BTT patients [19], the excellent durability of HM3 has made LVAD implantation as DT (Destination Therapy) a viable option. In recent years, there has been an increase in LVAD implantation for patients over 65 years old, despite associated increased mortality. Older patients are more commonly designated as DT candidates (61.1% in over 65-year-olds vs. 12.9% in 50–65-year-olds vs. 3.4% in those under 50) [22]. The 2022 INTERMACS registry also demonstrated an increased proportion of DT from 50.4% (2012–2016) to 66.4% (2017–2021), while the BTT proportion has declined from 27.5% to 19.5%. Although BTT has better survival rates due to younger age and fewer comorbidities compared to DT, 1-year survival in DT is still acceptable (77%) [23,24,25].

Regarding the changes in risk factors for LVAD implantation, from 2014 to 2018, predictors of mortality at one and two years after HM3 implantation included age, prior cardiac surgery, lower serum sodium, higher blood urea nitrogen, small left ventricular size, and right atrial pressure-to-pulmonary capillary wedge pressure ratio >0.6 [26]. In our study, being aged over 60 years remained a risk, but CKD, prior CABG surgery, and liver disease did not.

These previously known risk factors are still under debate. Nayak et al. analyzed 515 patients from the MOMENTUM3 trial and identified that prior cardiac surgery was associated with 5-year mortality [27]. However, a later propensity score-matched cohort, including 321 patients from 2006 to 2018, showed that redo sternotomy was not a risk factor for LVAD implantation [28]. Furthermore, Chou et al. investigated overall survival of continuous-flow LVAD implantation for patients with and without ischemic etiologies, revealing no difference between the two groups [29]. Furthermore, an analysis using 2012–2015 NIS data revealed that CKD stage 1–3 and stage 4–5 were associated with increased risk of in-hospital mortality (aOR: 1.33, CI 1.16–1.50; and 8.95, CI 6.90–11.61, respectively) [30]. However, a later comparative study of LVAD implantation for low versus high glomerular filtration rate (GFR) patients validated that the perioperative management, including inotrope support, mechanical circulation support, and optimizing volume status, for patients with GFR < 30 mL/min/1.73 m^2^ improved median GFR from 16 to 79 mL/min/1.73 m^2^ at discharge without worsening mid-term mortality [31]. Given the potential improvement of perioperative CKD management, future studies should include stratified CKD stage for analysis to interpret the genuine effect of CKD on LVAD implantation. Similarly, the association of diabetes mellitus type 2 and mortality for post-LVAD implantation also shows conflicting results [32,33]. Diabetes mellitus type 2 is a controllable disease to some degree; the proper stratification of severity should be incorporated in future studies. Lastly, liver disease was a previous risk factor, and our results showed it to be more prevalent in the younger generation. A study utilizing 2012–2017 NIS data for LVAD implantation demonstrated that patients with chronic liver disease were 52.8 ± 14.2 years old on average [34]. This trend may have continued in 2019 and 2020. Moreover, this study identified that patients with chronic liver disease had a higher OR of major bleeding (1.24, 95% CI 1.09–1.41). Younger age groups had a higher percentage of postoperative bleeding events in our data; thus, the association of liver disease and postoperative bleeding events should be further investigated using the updated dataset.

Although age is an independent risk of in-hospital mortality for LVAD implantation in our study, improved quality of LVAD can still serve as a therapeutic option for the older generation. The increased utilization of HM3 and worse functional status associated with older age in LVAD patients in the post-allocation era may imply that the role of LVADs has been shifting toward DT from BTT for older generations in the United States in the new allocation era.

### 4.4. LOS and Impact of Age 

Our research showed an inverse relationship between age and LOS, indicating that older individuals generally experienced shorter LOS. Specifically, age above 70 was identified as a negative predictor for LOS. In contrast, a study conducted by Cotts et al. showed that higher age posed a risk factor for extended hospital LOS [35]. The observed patterns in our results may be attributed to the higher mortality rates within this patient population, as indicated by the shorter LOS.

### 4.5. Increased Utilization of Nonhome Destination

Sanaiha et al., in their study using the NIS database, reported similar rates of nonhome discharge from 2008 to 2016 [36]. In contrast, our study showed an increase in nonhome discharge rates across all age categories. Managing LVAD requires cautious supervision, and utilizing nonhome services post-discharge is important [37]. Adequate cardiac rehabilitation, encompassing physical, occupational, and nutritional therapy, is integral for patients’ recovery, with some individuals requiring ongoing support for sustained self-care and independent living beyond the initial hospitalization period after LVAD implantation [37]. 

## 5. Limitation

As an observational, retrospective study, it is subject to inherent limitations. Moreover, the utilization of the NIS data may undermine the external generalizability of our findings. The data on comorbidities were treated as categorical variables, which may not accurately reflect the effects of disease severity on LVAD implantation. Another limitation is that NIS data do not report events in a chronological fashion, making it challenging to isolate potential preoperative and intraoperative complications.

It is important to recognize that NIS data are 1-year data without outpatient follow-up capabilities, which poses challenges in tracking long-term outcomes. Furthermore, the database’s limitations make it difficult to fully account for variables that may influence results, such as lifestyle and quality of care. While we have incorporated available baseline characteristics like socioeconomic status and geographical areas, inherent limitations persist. Future studies may be necessary to comprehensively understand the impact of lifestyle and quality of care on both short- and long-term outcomes post-LVAD implantation. Additionally, we acknowledge that grouping patients over the age of 70 may hide significant differences within that age group, but our decision was driven by the aim to maintain nearly equal distributions across all age groups and maintain statistical power.

Finally, there is a possibility of overestimating the actual number of LVAD implantations by weighing in the analysis; however, the percentages and regression analyses are accurate.

## 6. Conclusions

In-hospital mortality rates of patients who underwent LVAD implantation from 2019 to 2020 were 7%, 6%, 12%, and 17% in the 18–49, 50–59, 60–69, and over 70 age groups, respectively. Independent predictors of in-hospital mortality included being over 60 years old and higher Charlson Comorbidity Index. Additionally, a shorter LOS was seen in the over-70 age group. The predictors of shorter LOS were aged over 70, west region, and urban teaching facility. The total hospitalization charges had not shown a statistical significance across the age groups. However, the utilization of nonhome services on discharge was statistically higher across all age categories. Our study highlights age-related outcomes and emphasizes the importance of considering age in LVAD patient selection and management strategies. While our study highlights the association between older age and mortality in the LVAD population, we stress that age alone should not be viewed as a contraindication for LVAD. We recommend holding thorough discussions with patients, considering the advanced comorbidities and impairment often present in the end-stage heart failure population.

## Figures and Tables

**Table 1:** Characteristics of left ventricular assist device patients among age categories.

	18–49	50–59	60–69	≥70	*p*-Value
Total (n = 7375)	1935	1900	2300	1240	
Mean Age	38	55	64	73	0.000
Male	73%	72%	75%	85%	0.003
Race					
White	40%	57%	61%	80%	0.000
Black	44%	32%	27%	14%	
Hispanic	8%	7%	5%	4%	
Asian or Pacific Islander	3%	1%	2%	1%	
Insurance					
Medicare	28%	32%	62%	88%	0.000
Medicaid	32%	18%	8%	0.0%	
Private	37%	47%	30%	11%	
Self-pay	3%	2%	0.00%	0.00%	
Median household income					
$1–$49,999	37%	29%	26%	21%	0.000
$50,000–$64,999	24%	31%	30%	24%	
$65,000–85,999	20%	24%	27%	26%	
$86,000 or more	19%	16%	17%	28%	
Hospital Region					
Northeast	18%	17%	20%	26%	0.143
Midwest	27%	28%	27%	24%	
South	44%	43%	42%	35%	
West	11%	11%	11%	15%	
Relative bed size category of hospital					
Small	2%	3%	2%	7%	0.008
Medium	6%	6%	9%	10%	
Large	92%	91%	89%	83%	
Location/teaching status of hospital					
Rural	N/A	N/A	N/A	N/A	0.054
Urban non-teaching	0%	1%	0%	2%	
Urban teaching	100%	99%	100%	98%	
Charlson Comorbidity Index					
0	0%	0%	0%	1%	0.000
1	20%	14%	9%	10%	
2	21%	14%	13%	12%	
≥3	59%	71%	78%	78%	
Hypertension	2%	1%	1%	2%	0.924
Chronic heart failure	97%	97%	99%	98%	0.143
Coronary artery disease	28%	52%	63%	62%	0.000
Dyslipidemia	19%	34%	36%	37%	0.000
Previous myocardial infarction	9%	16%	17%	14%	0.011
Chronic obstructive pulmonary disease	18%	22%	20%	18%	0.469
Liver Disease	14%	12%	9%	8%	0.019
Chronic kidney disease	9%	7%	7%	10%	0.281
Peripheral vascular disease	5%	8%	12%	15%	0.000
Previous coronary artery bypass grafting	1%	5%	10%	10%	0.000
Smoking	27%	24%	27%	22%	0.473

**Table 2:** Inpatient outcomes of left ventricular assist devices implantation among age categories.

	18–49	50–59	60–69	≥70	*p*-Value
In-hospital mortality	140 (7%)	115 (6%)	265 (12%)	215 (17%)	0.000
Non-elective admission	405 (21%)	500 (26%)	665 (29%)	435 (35%)	0.000
Driveline infection	125 (6%)	70 (4%)	100 (4%)	35 (3%)	0.1
Acute kidney injury	1425 (74%)	1335 (70%)	1765 (77%)	940 (76%)	0.169
Acute kidney injury requiring dialysis	205 (11%)	190 (10%)	180 (8%)	105 (8%)	0.58
Ischemic stroke	70 (4%)	110 (6%)	105 (5%)	80 (6%)	0.325
Postoperative bleeding	390 (20%)	395 (21%)	570 (25%)	170 (14%)	0.005
Intracranial hemorrhage	60 (3%)	40 (2%)	80 (3%)	25 (2%)	0.55
Gastrointestinal bleeding	170 (9%)	200 (11%)	310 (13%)	100 (8%)	0.06
Hemoptysis	85 (4%)	55 (3%)	105 (5%)	25 (2%)	0.23
Hematuria	45 (2%)	45 (2%)	60 (3%)	10 (1%)	0.47
Unspecified bleeding	75 (4%)	85 (4%)	100 (4%)	20 (2%)	0.39
Epistaxis	0 (0.00%)	10 (0.01%)	5 (0.00%)	0 (0.00%)	0.24
Bleeding requiring transfusion	105 (5%)	110 (6%)	145 (6%)	30 (2%)	0.17
Septic shock	170 (9%)	160 (8%)	170 (7%)	70 (6%)	0.415

**Table 3:** Trends of outcomes in left ventricular assist device patients among age categories.

Variables	Year	18–49	*p*-Value	50–59	*p*-Value	60–69	*p*-Value	≥70	*p*-Value
In-hospital mortality	2019	5%	0.1	6.3%	0.84	11.1%	0.77	16.1%	0.6
	2020	9.6%		6%		12%		18.5%	
Acute kidney injury requiring dialysis	2019	8%	0.14	8.7%	0.4	6%	0.14	4%	0.02
	2020	13.3%		11.5%		10%		13%	
Intracranial hemorrhage	2019	3%	0.92	2.4%	0.62	3.1%	0.7	2.4%	0.65
	2020	3.2%		1.7%		3.8%		1.6%	
Gastrointestinal bleeding	2019	7.5%	0.36	10.2%	0.83	14.3%	0.58	9.6%	0.33
	2020	10.1%		10.9%		12.5%		6.5%	
Postoperative bleeding	2019	19.1%	0.59	19.9%	0.66	25.8%	0.59	15.3%	0.45
	2020	21.3%		21.8%		23.6%		12.1%	
Ischemic stroke	2019	3.5%	0.91	4.4%	0.21	5%	0.83	8.1%	0.28
	2020	3.7%		7.5%		4.3%		5%	
Driveline infection	2019	4.5%	0.08	3.4%	0.73	5%	0.63	4%	0.25
	2020	8.6%		4%		4%		2%	
Septic shock	2019	8%	0.33	6.3%	0.11	6.7%	0.57	4%	0.25
	2020	10%		11%		8.2%		7.3%	

*p*-values are for trend analysis.

**Table 4:** Independent predictors of mortality in left ventricular assist device patients.

Variable	Adjusted Odds Ratio	*p*-Value	Confidence Interval
Age category			
50–59	0.89	0.76	0.44–1.82
60–69	1.99	0.02	1.12–3.57
≥70	2.88	0.002	1.45–5.71
Female	0.97	0.88	0.64–1.47
Race			
Black	1.01	0.97	0.63–1.63
Hispanic	0.78	0.57	0.32–1.87
Asian or Pacific Islander	1.75	0.32	0.58–5.29
Native American	1.77	0.59	0.22–14.37
Other	1.01	0.98	0.35–2.92
Elective admission	0.65	0.08	0.4–1.05
Hospital region			
Midwest	1.16	0.63	0.63–2.13
South	1.09	0.76	0.64–1.86
West	1.07	0.83	0.57–2.0
Teaching hospital	0.32	0.14	0.07–1.44
Hospital bed size			
Medium	0.56	0.42	0.14–2.26
Large	0.97	0.96	0.31–3.01
Insurance			
Medicaid	0.99	0.99	0.56–1.76
Private	1.1	0.67	0.71–1.70
Self-pay	0.52	0.53	0.06–4.1
Income			
$50,000–64,999	0.87	0.6	0.52–1.46
$65,000–85,999	0.98	0.94	0.58–1.64
$86,000 or more	1.46	0.19	0.83–2.55
Hypertension	1.52	0.53	0.41–5.62
Chronic obstructive lung disease	0.58	0.07	0.32–1.05
Chronic kidney disease	0.3	0.03	0.1–0.9
Coronary artery disease	0.6	0.03	0.39–0.94
Previous myocardial infarction	0.15	0.001	0.05–0.47
Diabetes mellitus type 2	0.59	0.01	0.4–0.88
Obesity	1.21	0.47	0.72–2.06
Peripheral vascular disease	0.81	0.58	0.38–1.71
Liver disease	0.53	0.12	0.24–1.18
Previous coronary artery bypass grafting	1.1	0.86	0.44–2.7
Charlson Comorbidity Index	1.39	0.02	1.05–1.84

Obesity is defined as body mass index ≥ 30.

**Table 5:** Mean length of stay of left ventricular assist device patients stratified by age category.

Age Category	Mean Length of Stay	SD	*p*-Value
18–49	39.1	36.3–41.9	
50–59	37.7	34.9–40.6	<0.01
60–69	36.5	33.9–39.1	
≥70	31.9	29.2–34.5	

SD; standard deviation.

**Table 6:** Total hospitalization charge of left ventricular assist device patients among age categories.

Age Category	Mean Total Charge (10^5^)	SD	*p*-Value
18–49	1.25	1.13–1.35	
50–59	1.14	1.04–1.23	0.23
60–69	1.17	1.09–1.26	
≥70	1.11	1.01–1.21	

SD; standard deviation.

## Data Availability

The data utilized in this research are available from the author upon request.
